# Exploring the role of the social vulnerability index in understanding COVID-19 immunization rates

**DOI:** 10.1371/journal.pone.0302934

**Published:** 2024-06-07

**Authors:** Lung-Chang Chien, Erika Raquel Marquez, Samantha Smith, Tiana Tu, Amanda Haboush-Deloye

**Affiliations:** 1 Department of Epidemiology and Biostatistics, University of Nevada Las Vegas School of Public Health, Las Vegas, Nevada, United States of America; 2 Department of Environmental and Occupational Health, University of Nevada Las Vegas School of Public Health, Las Vegas, Nevada, United States of America; 3 Nevada Institute for Children’s Research and Policy, Las Vegas, Nevada, United States of America; University of Arizona Medical Center - University Campus: Banner University Medical Center Tucson, UNITED STATES

## Abstract

Communities that are historically marginalized and minoritized were disproportionately impacted by the COVID-19 pandemic due to long-standing social inequities. It was found that those who experience social vulnerabilities faced a heightened burden of COVID-19 morbidities and mortalities and concerningly lower rates of COVID-19 vaccination. The CDC’s Social Vulnerability Index (CDC-SVI) is a pivotal tool for planning responses to health crises such as the COVID-19 pandemic. This study explores the associations between CDC-SVI and its corresponding themes with COVID-19 vaccine uptake in Nevada counties. Additionally, the study discusses the utility of the CDC-SVI in the context of equitable vaccine uptake in a pandemic setting. We examined the linear association between the 2020 CDC-SVI (including the composite score and the four themes) and COVID-19 vaccine uptake (including initial and complete vaccinations) for the seventeen Nevada counties. These associations were further examined for spatial-varied effects. Each CDC-SVI theme was negatively correlated with initial and complete COVID-19 vaccine uptake (crude) except for minority status, which was positively correlated. However, all correlations were found to be weak. Excessive vaccination rates among some counties are not explained by the CDC-SVI. Overall, these findings suggest the CDC-SVI themes are a better predictor of COVID-19 vaccine uptake than the composite SVI score at the county level. Our findings are consistent with similar studies. The CDC-SVI is a useful measure for public health preparedness, but with limitations. Further understanding is needed of which measures of social vulnerability impact health outcomes.

## Introduction

Communities that have been historically and intentionally excluded were disproportionately burdened by the COVID-19 pandemic [1,2]. These communities consist of those historically marginalized and minoritized for social characteristics like race and ethnicity, gender, sexual orientation, or level of education [3], and are often underrepresented and underserved in both health care and social services. As a result of this historical and intentional exclusion, many of these communities persistently experience poorer health outcomes, which are also associated with elevated COVID-19 morbidities and mortalities [4–6]. Vaccines play a critical role in protecting individual and community health from infectious diseases by providing immunity, preventing the spread, limiting the severity of illness, and reducing mortality rates [7]. When the COVID-19 vaccines became available to the public in December 2020, communities disproportionately impacted by the pandemic were the least likely to be vaccinated [8,9].

The history of vaccine-preventable diseases has presented a strong inverse association between groups that have been socially marginalized and vaccination rates [8,10]. More specifically, these groups tend to experience more barriers to vaccine access and acceptance that stem from a range of factors such as limited access to healthcare, lower healthcare fluency, geographical location, and low trust in medicine [11–13]. Socially marginalized groups have faced exploitation by the medical community (e.g., the 1932 Tuskegee Syphilis study), which has exacerbated the ongoing distrust of the healthcare system [14]. The Centers for Disease Control and Prevention (CDC) reported in March 2021 that United States (U.S.) counties that experience greater social vulnerability had lower COVID-19 vaccination rates (13.9%) when compared to counties that experience less social vulnerability (15.8%) [15]. Another study analyzed COVID-19 vaccine distributions from the beginning of vaccine rollout within two major cities which found distribution inequities across levels of social vulnerability, communities based on racial and ethnic background, and types of vaccination site [16]. Even though some COVID-19 vaccine distribution efforts were able to reach communities that are made vulnerable, there continue to be many barriers and challenges to achieving vaccine equity which implies the need for fair and just access to vaccines [13]. Ultimately, to achieve this ideal and mitigate the disproportionate impacts of COVID-19, it is essential to equitably distribute the vaccines.

One tool used to bridge the gap in vaccine equity during the COVID-19 pandemic is the CDC’s Social Vulnerability Index (CDC-SVI) [17,18]. In 2011, CDC-SVI was created as a measure for public health officials and emergency responders to use during crises—such as pandemics—to prioritize the allocation of resources and assistance to communities that are in the most need [1]. The CDC-SVI can also be employed to plan vaccine allocation efforts by using it in conjunction with a geographic information system (GIS) and geospatial analysis. A combination of the CDC-SVI and GIS can inform the planning of on-the-ground efforts and distribution of vaccines [19–21]. This paper aims to assess the association between the CDC-SVI and corresponding themes with COVID-19 vaccine uptake. In particular, we hypothesized that COVID-19 vaccine uptake was unequally distributed among counties. We also hypothesized that the CDC-SVI’s themes unequally contributed to the index, implying that their impacts on COVID-19 vaccine uptake may vary. The ultimate goal of this study is to present more details about the influence of the CDC-SVI in a given area and examine methodological concerns about the utilization of the CDC-SVI for future research.

## Methods

### Study area

**Fig 1:**
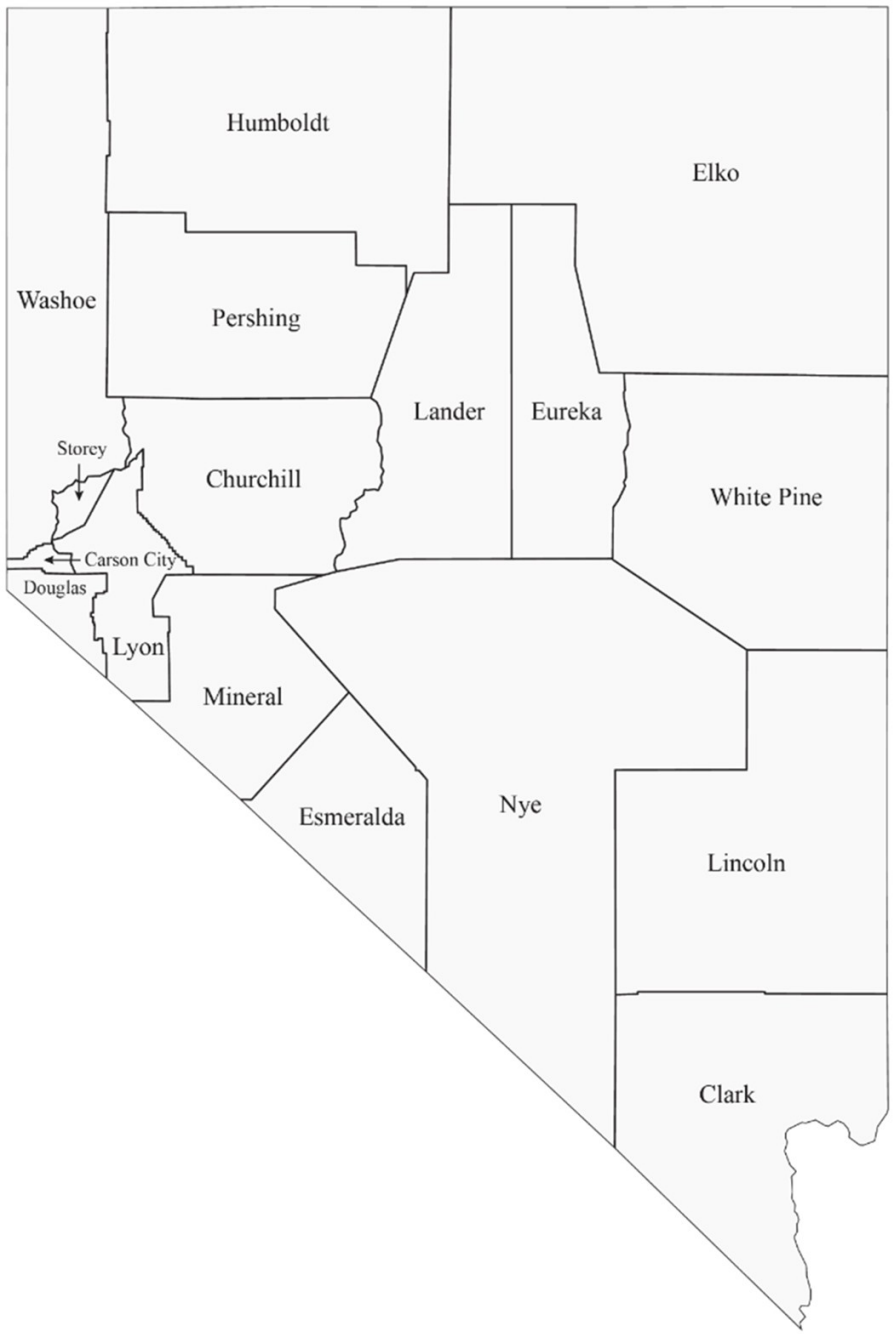
Counties in Nevada.

The population of Nevada is comprised of 3.1 million people across 17 counties (Fig 1) [22]. The state is the sixth fastest-growing and is among the nation’s top ten most racially and ethnically diverse [23,24]. Among Nevada’s counties, Clark County is the most populated (2.3 million), making up almost 73% of the state’s total population count, and is the 22^nd^ most diverse county in the U.S. [22,25,26].

### Data collection

To examine the relationship between COVID-19 vaccine coverage and social vulnerability, we collected vaccine coverage data by county (n = 17) from the Nevada Department of Health and Human Services. The data was gathered on a weekly basis from April 19, 2021, to April 5, 2022.

The latest CDC-SVI data in 2020 at the county level in Nevada was computed from 16 social factors grouped into four themes, according to the CDC-SVI methods (Table 1) [27]. The raw data were gathered from the 2016–2020 5-year estimates from the American Community Survey (ACS), which were transformed into percentile percentages and summed for each theme. The sums of the themes were then combined to form the CDC-SVI score with equal weight to each theme. Counties with a higher score are considered to have higher vulnerability.

**Table 1:** CDC social vulnerability index variables.

Theme	Social Factor
1. Socioeconomic Status	Below 150% poverty Unemployed Housing cost burden No high school diploma No health insurance
2. Household Characteristics	Aged 65 or older Aged 17 or younger Civilian with a disability Single-parent households English language proficiency
3. Minority Status	• Hispanic or Latino (of any race); Black and African American, Not Hispanic or Latino; American Indian and Alaska Native, Not Hispanic or Latino; Asian, Not Hispanic or Latino; Native Hawaiian and Other Pacific Islander, Not Hispanic or Latino; Two or More Races, Not Hispanic or Latino; Other Races, Not Hispanic or Latino
4. Housing Type and Transportation	Multi-unit structures Mobile homes Crowding (more than 1 person per room) No vehicle Group Quarters

### Data analysis

This study first examined the linear association between CDC-SVI (including CDC-SVI itself and the four themes) and COVID-19 initial and complete vaccinations, adjusted by spatial effects. We defined initial vaccine coverage as the cumulative percentage of the population who started a COVID-19 vaccine series (received only a first dose of either Pfizer-BioNTech or Moderna vaccine), not including COVID-19 boosters. Complete vaccination coverage was defined as the cumulative percentage of the population who completed a COVID-19 vaccine series (received two doses of Pfizer-BioNTech or Moderna, or one dose of Johnson & Johnson/Janssen vaccine), not including COVID-19 boosters. Then, we further examined the spatial-varied effects between CDC-SVI and COVID-19 vaccination to identify areas with a high CDC-SVI leading to a high vaccination rate. Because these analyses involved spatial functions, which cannot be well adopted in traditional models, we instead applied the Bayesian additive model to fulfill our study aims [28]. By defining *Y*_*it*_ as the number of people with initial or complete vaccination in the *i*^th^ county (*i* = 1, …, 17) on the *t*^th^ week (*t* = 1, …, 53), it follows a Poisson distribution with a mean parameter *μ*_*it*_, which can be expressed as:

Model 1:

$$log\;log\;\left(\mu_{it}\right)=\alpha +\sum_{j=1}^{4}\beta_{j}\times SVI\_THEME_{ij}+f\left(t\right)+f\left(spatial\right)+offset$$

Model 2:

$$log\;log\;\left(\mu_{it}\right)=\alpha +\beta \times SVI_{i}+f\left(t\right)+f\left(spatial\right)+offset$$

where *α* is the intercept, and *β*_*j*_ and *β* are the coefficients of the *j*^th^ theme and CDC-SVI itself. The term *f*(*t*) is a nonlinear function with a penalized smoothing basis and 6 interior knots for the calendar time *t*. The term *f*(*spatial*) is a spatial function estimated by Markov random fields, which derived a neighborhood matrix to estimate the spatial coefficient [29]. When the spatial function is an independent term in the model, like Model 1, the spatial estimate can be explained as the excessive rate of COVID-19 vaccination unexplained by the themes and CDC-SVI itself. The last term is an offset from the logarithm of the 2020 population at the county level.

To further investigate the association between the themes or CDC-SVI and vaccination rate varied by county, we replaced linear terms with interaction terms between each theme or CDC-SVI and the spatial function in Model 1 and Model 2, expressed as:

Model 3:

$$log\;log\;\left(\mu_{it}\right)=\alpha +\sum_{j=1}^{4}SVI\_THEME_{ij}\times f_{j}\left(spatial\right)+f\left(t\right)+offset$$

Model 4:

$$log\;log\;\left(\mu_{it}\right)=\alpha +SVI_{i}\times f\left(spatial\right)+f\left(t\right)+offset$$

All model settings in Model 3 and Model 4 are identical to those in Model 1 and Model 2. All unknown parameters were estimated through a Bayesian inference based on Markov chain Monte Carlo (MCMC) simulations. The posterior distribution of each unknown parameter was first built by 50,000 iterations, where the first 10,000 iterations were burn-in, and a sample was drawn every 40 iterations. The mean of the posterior distribution represented the point estimate of an unknown parameter. Based on the significance level of 0.05, the 2.5^th^ and 97.5^th^ percentiles of the posterior distribution composed the 95% credible interval (CI) of a point estimate. Thus, a significant estimate was determined by its 95% CI strictly larger or smaller than 0. We also utilized the deviance information criterion (DIC) to compare models.

The estimated coefficients of linear predictors or spatial estimates are equivalent to the logarithm of relative risk (logRR), which was transformed into RR% to facilitate epidemiological explanations. The 95% CI of RR% has also transformed accordingly. All models were diagnosed through worm plots, trace plots, and autocorrelation function plots for accessing the fit of our models, the robustness of resampling in MCMC, and the autocorrelation, respectively. Sensitivity analysis was conducted with another two sets of hyperparameters in terms of (1, 0.05) and (0.001, 0.001). We applied the repeated measures ANOVA to compare them with the original results.

Data were cleaned and summarized by SAS v9.4 (SAS Institute Inc., Cary, NC). Spatial data analysis and mapping were accomplished by RStudio v2022.07.2+576 (RStudio Team, Boston, MA). The significant level was set to 5%.

## Results

### Vaccinations and CDC-SVI by county

**Fig 2:**
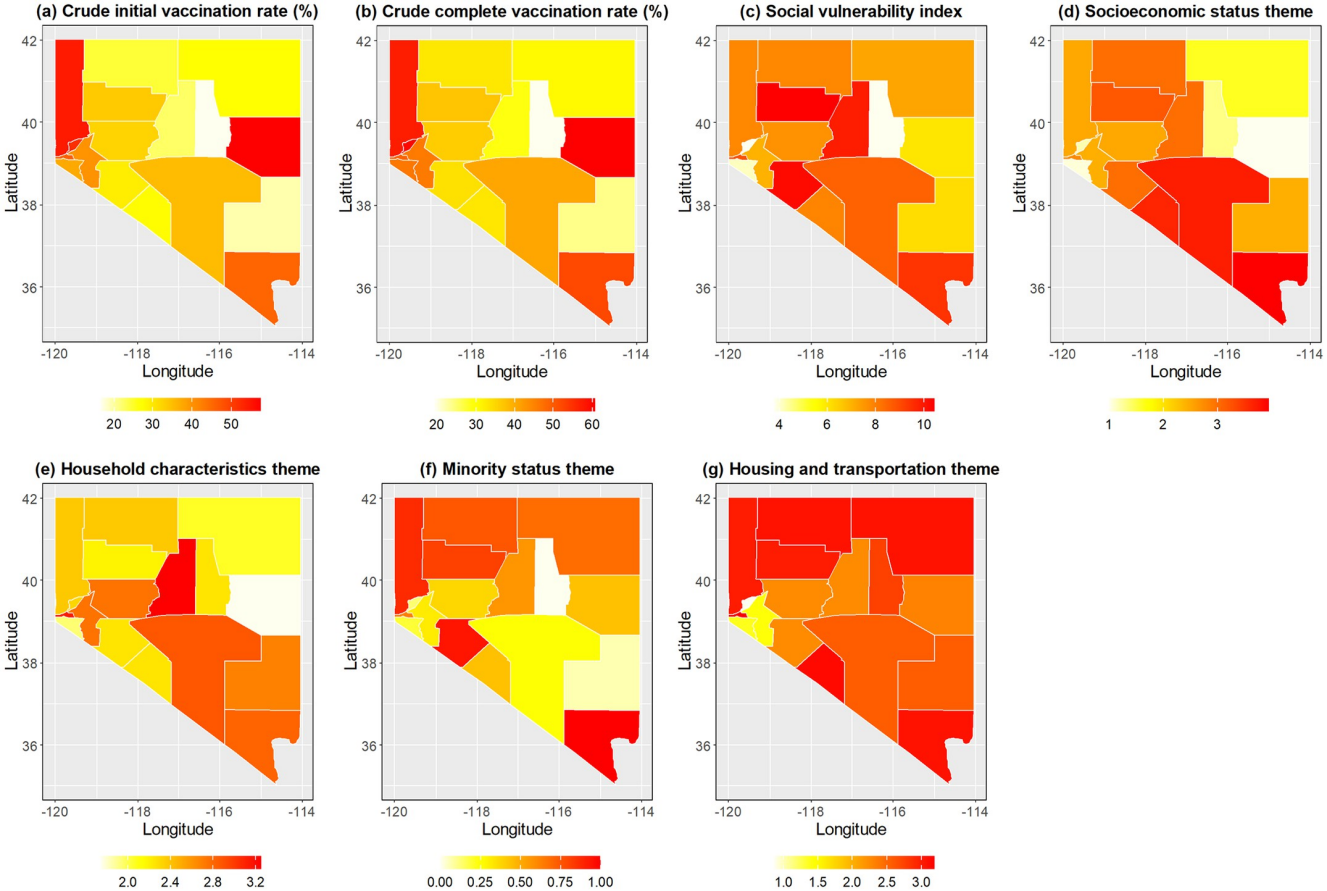
Spatial distributions of COVID-19 vaccination rate and social vulnerability index with four themes at the county level in Nevada.

Fig 2 shows geographic patterns of COVID-19 vaccination and CDC-SVI measures in Nevada counties in 2021. The crude initial vaccination rate ranged from 16.72% to 57.65% (Mean = 35.41; SD = 12.40). The crude complete vaccination rate ranged from 19.58% to 60.83% (Mean = 40.56; SD = 12.47). Both rates were high in Washoe County, Elko County, Storey County, and Clark County (Fig 2a and 2b). The CDC-SVI ranged from 4.50 to 10.82 (Mean = 7.93; SD = 1.78). The geospatial patterns of the four themes can be referred to in Fig 2d–2g. Table 2 shows that among the four themes, the highest average was 2.49 (SD = 0.94) in the socioeconomic status theme, whereas the minority theme has the lowest average of 0.05 (SD = 0.32). The theme proportions further reveal that, on average, the housing characteristics theme has the largest proportion representing 32.38% (SD = 7.35) in the SVI. In contrast, the minority theme has the smallest proportion with an average of 5.97% (SD = 3.29) in the CDC-SVI. We also found that the highest theme score appeared differently in different counties, implying that a theme with a higher proportion still displays an equal role to a theme with a lower proportion because the CDC-SVI was computed by an immediate summation.

**Table 2:** Theme scores and proportions in the CDC-SVI by Nevada county.

County	Socioeconomic Status		Household Characteristics		Minority Status		Housing and Transportation	
	Score	Proportion	Score	Proportion	Score	Proportion	Score	Proportion
Carson City	2.75	28.39	3.13	32.26	0.63	6.45	3.19	32.90
Churchill	2.50	31.75	2.75	34.92	0.38	4.76	2.25	28.57
Clark	3.94	36.42	2.81	26.01	1.00	9.25	3.06	28.32
Douglas	1.06	23.29	1.94	42.47	0.19	4.11	1.38	30.14
Elko	1.63	21.85	2.06	27.73	0.69	9.24	3.06	41.18
Esmeralda	3.63	38.41	2.25	23.84	0.44	4.64	3.13	33.11
Eureka	1.31	20.79	2.25	35.64	0.00	0.00	2.75	43.56
Humboldt	3.00	32.65	2.38	25.85	0.75	8.16	3.06	33.33
Lander	3.00	33.10	3.25	35.86	0.56	6.21	2.25	24.83
Lincoln	2.44	31.45	2.69	34.68	0.06	0.81	2.56	33.06
Lyon	2.44	35.14	2.75	39.64	0.31	4.50	1.44	20.72
Mineral	3.00	35.56	2.25	26.67	0.94	11.11	2.25	26.67
Nye	3.69	39.33	2.88	30.67	0.25	2.67	2.56	27.33
Pershing	3.19	34.69	2.19	23.81	0.81	8.84	3.00	32.65
Storey	1.19	26.39	2.31	51.39	0.13	2.78	0.88	19.44
Washoe	2.50	28.57	2.38	27.14	0.88	10.00	3.00	34.29
White Pine	1.00	18.18	1.75	31.82	0.44	7.95	2.31	42.05
Mean	2.49	30.35	2.47	32.38	0.50	5.97	2.48	31.30
SD	0.94	6.38	0.42	7.35	0.32	3.29	0.69	6.78

Table 3 shows the correlation coefficients among vaccination, CDC-SVI, and its themes. The CDC-SVI was negatively correlated with both vaccination rates. Among the four themes, socioeconomic status, household characteristics, and housing and transportation themes were negatively correlated with both vaccination rates. Minority status is the only theme positively correlated with both vaccination rates. All correlations between vaccination rates and themes were weak. The highest correlation among the four themes was 0.57 (95% CI = 0.10, 0.82) between socioeconomic status and household characteristics. The CDC-SVI itself is highly correlated with the socioeconomic status theme by 0.82 (95% CI = 0.54, 0.93).

**Table 3:** Pearson correlations among crude vaccination rates and social vulnerability index themes. Parentheses show the 95% confidence intervals.

	Complete vax	Theme 1	Theme 2	Theme 3	Theme 4	SVI
Initial vax	0.99 (0.96, 0.99)	-0.19 (-0.61, 0.32)	-0.14 (-0.58, 0.37)	0.21 (-0.30, 0.63)	-0.24 (-0.64, 0.28)	-0.07 (-0.53, 0.42)
Complete vax		-0.18 (-0.61, 0.33)	-0.14 (-0.58, 0.37)	0.19 (-0.32, 0.61)	-0.32 (-0.69, 0.20)	-0.11 (-0.56, 0.40)
Theme 1			0.57 (0.10, 0.82)	0.52 (0.03, 0.79)	0.52 (0.04, 0.80)	0.82 (0.54, 0.93)
Theme 2				0.04 (-0.45, 0.51)	0.11 (-0.40, 0.56)	0.44 (-0.06, 0.76)
Theme 3					0.52 (0.04, 0.80)	0.77 (0.45, 0.91)
Theme 4						0.52 (0.04, 0.80)

Initial vax = Crude initial vaccination rate; Complete vax = Crude complete vaccination rate; Theme 1 = Socioeconomic status; Theme 2 = Household characteristics; Theme 3 = Minority status; Theme 4 = Housing and transportation; SVI = Social vulnerability index.

### Measuring linear associations between CDC-SVI, themes, and vaccinations for the state

**Fig 3:**
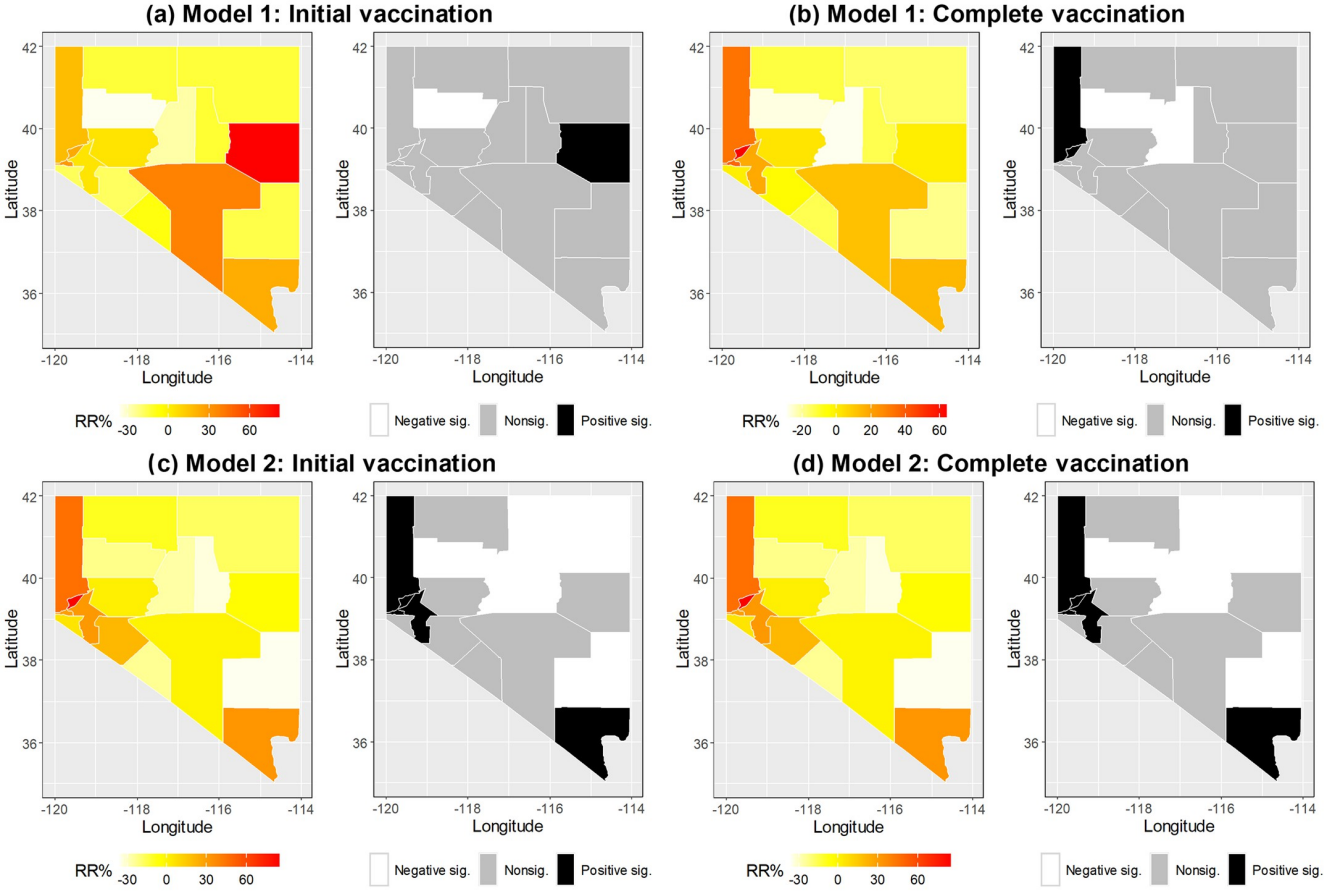
Comparisons of relative rate percentages obtained from the spatial function in Model 1 and Model 2. Significance was determined by the 95% credible interval in each county.

Table 4 shows the linear relationship between themes and vaccination from the first two models. In Model 1, only the minority status theme was significantly associated with complete vaccination with an RR% of 71.68 (95% CI = 1.46, 180.82), but no significant association was found for initial vaccination. In Model 2, the CDC-SVI was not significantly related to both initial and complete vaccinations. Regardless of initial vaccination or complete vaccination, Model 1 has a smaller DIC than Model 2, indicating that the four themes performed better than the CDC-SVI. Fig 3 shows the spatial pattern with respect to the estimated spatial function in Model 1 and Model 2, revealing that, even though those patterns looked similar between the two models in either initial or complete vaccination, Model 2 had more significant counties than Model 1. Fig 3 also reveals that the vaccination rate was not completely explained by themes as linear terms in the first two models. More counties have either positively or negatively significant spatial estimates in Model 2 than in Model 1, indicating their initial and complete vaccination rates were excessively higher or lower than the state average. Overall findings indicate that the themes predict initial and complete vaccination rates better than the CDC-SVI itself, but the only significant spatial estimate was the minority status theme. In addition, positively significant counties more likely appeared in southern and western Nevada, indicating that these areas had excessive vaccination rates not explained by the CDC-SVI and its four themes.

**Table 4:** Associations between COVID-19 vaccination and CDC-SVI measures.

	Initial Vaccination			Complete Vaccination		
Variable	RR%	95% CI		RR%	95% CI	
Model 1	DIC = 14059338			DIC = 13423872		
Socioeconomic Status	-5.04	-34.08	39.59	2.08	-21.79	31.50
Household Characteristics	1.81	-45.50	81.86	0.29	-38.06	56.05
Minority Status	95.65	-4.27	297.95	71.68*	1.46	180.82
Housing and Transportation	-19.49	-44.83	15.89	-17.65	-35.39	7.64
Model 2	DIC = 14162493			DIC = 13570409		
SVI	-0.60	-10.23	10.66	1.16	-7.32	10.24

* Statistically significant at the 95% CI strictly different from 0.

Abbreviation: RR% = Relative rate percentage; CI = Confidence interval; DIC = Deviance information criteria; SVI = Social vulnerability index.

### Measuring interactions between CDC-SVI, themes, and vaccinations by county

**Fig 4:**
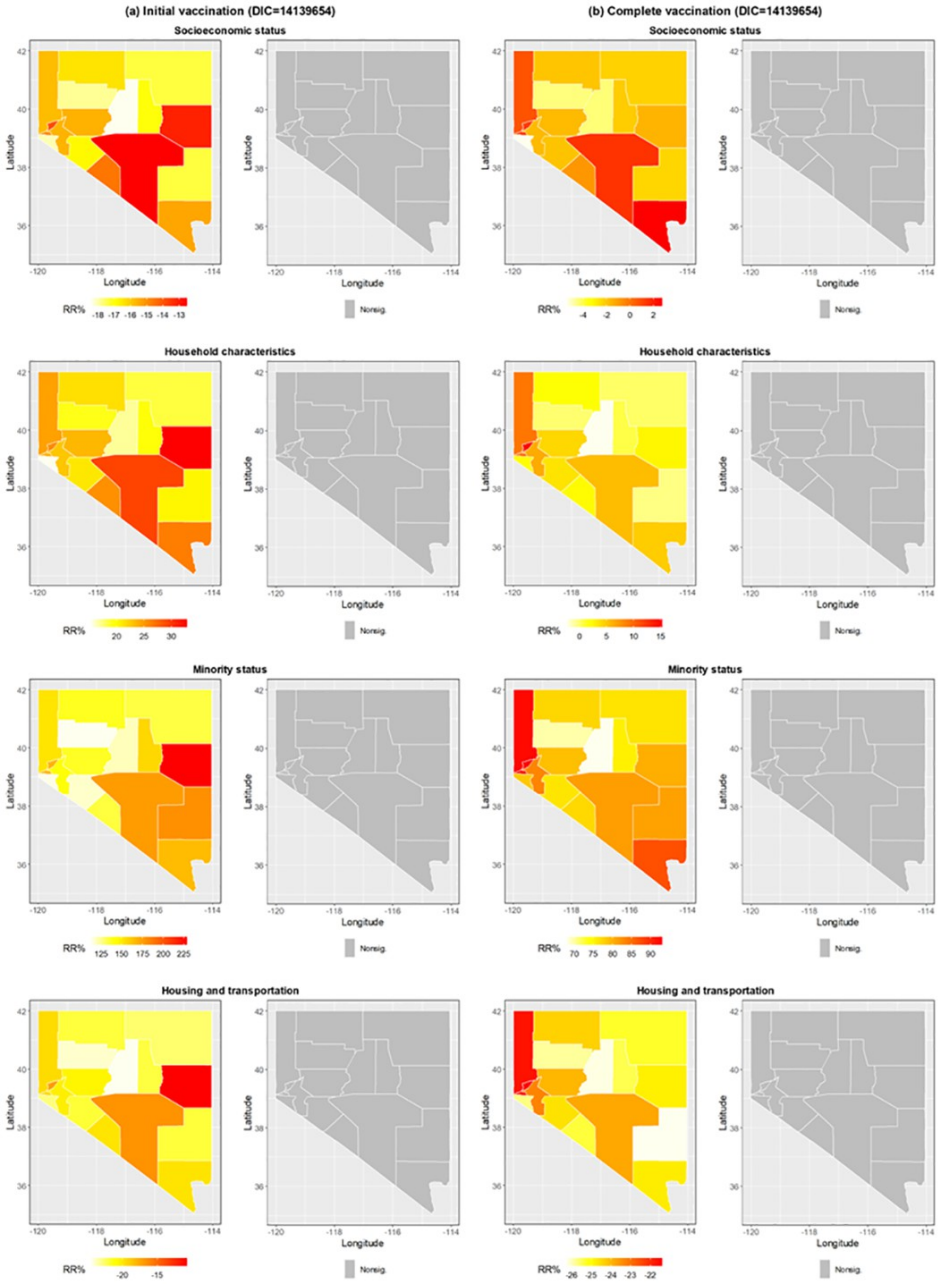
Relative rate percentages obtained from the spatial function in Model 3. Significance was determined by the 95% credible interval in each county.

**Fig 5:**
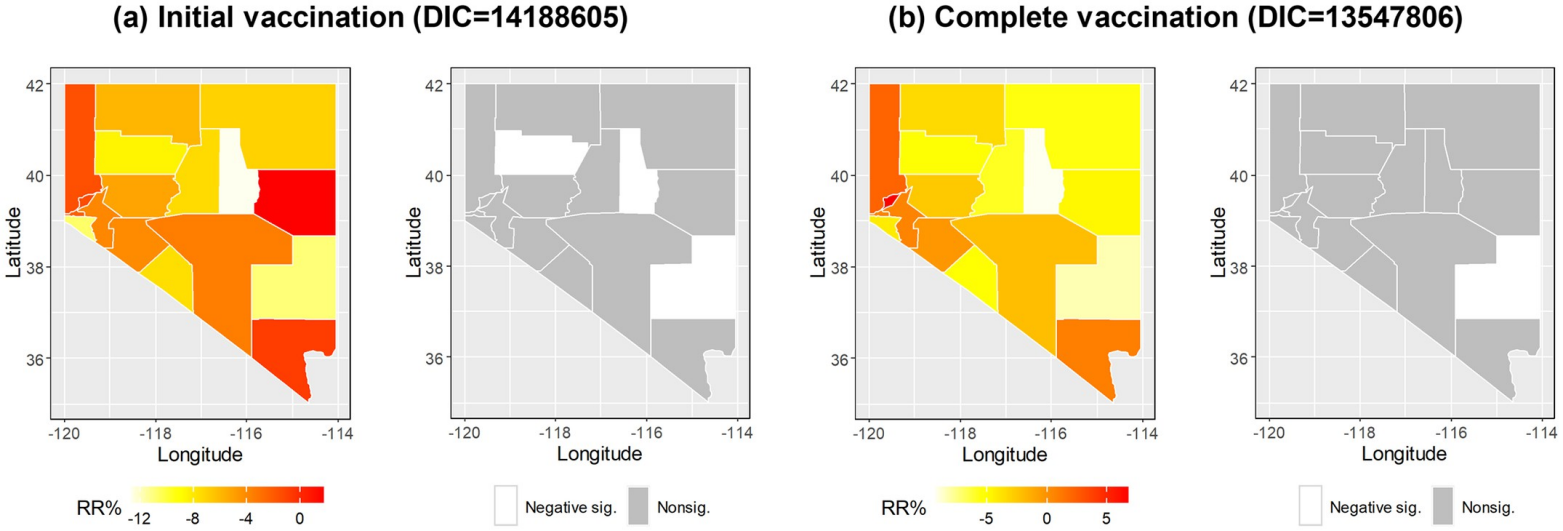
Relative rate percentages obtained from the spatial function in Model 4. Significance was determined by the 95% credible interval in each county.

When interacting with the spatial function in Model 3, the four themes display very different patterns, revealing that their influence on COVID-19 vaccination varied by county. Fig 4a shows that the RR% for initial vaccination was positive in all counties with respect to household characteristics and minority status themes, indicating that higher scores of household characteristics and minority status lead to the increase in COVID-19 vaccination rates in each county. On the contrary, socioeconomic status and housing and transportation themes were negatively associated with COVID-19 vaccination in all counties. However, none of them were statistically significant. Similarly, Fi 4b shows that the association between each theme and COVID-19 vaccination varied by county, but none were statistically significant. Compared to Model 1, where the four themes were linear predictors, Model 3 had worse performance with a much higher DIC for both initial vaccination (14059338 vs. 14139654) and complete vaccination (13423872 vs. 13454926). When the CDC-SVI itself interacted with the spatial function in Model 4, 16 of 17 counties had a negative RR% for initial vaccination, including three significant findings in Eureka County, Lincoln County, and Pershing County (Fig 5a). For complete vaccination, 12 of 17 counties had a negative RR%, whereas only Lincoln County had a significant association (Fig 5b). Compared to Model 2, where the CDC-SVI was defined as a linear predictor, Model 4 had a worse performance with a much higher DIC for initial vaccination (14162493 vs. 14188605). Nonetheless, Model 4 performed better than Model 2 for complete vaccination with a lower DIC (13570409 vs. 13547806).

## Discussion

The COVID-19 pandemic exposed and exacerbated deep-rooted health and social inequities worldwide, taking a disproportionately heavy toll on communities that are under-resourced. Therefore, developing vaccine equity and resource allocation strategies to identify communities that are historically socially vulnerable could not have been more urgent. This study was performed to determine how well the CDC-SVI can be used to explain vaccine uptake. The findings from our study indicate that the total CDC-SVI score was not a good predictor of COVID-19 incomplete or complete vaccinations, but certain themes may be better predictors at the county level.

To tackle disparities and the health impacts of emergencies, it is crucial to address the social determinants that contribute to poor health outcomes. In recent studies, researchers have aimed to better understand the connections between vulnerability factors, social vulnerability indices, and outcomes related to disasters and emergencies. The findings from this study are consistent with similar studies that have examined these associations. For instance, Karaye and Horney [4] examined the association between CDC-SVI and COVID-19 case counts and found that “SVI variables explained only 38.9% (R^2^ = 0.389) of the variability in COVID-19 case counts.” Nayak et al. [5] found no significant association between the overall CDC-SVI and COVID-19 incidence, whereas COVID-19 case fatality rates were associated with socioeconomic status and minority status themes. Another study examining social vulnerability and influenza vaccinations among Medicare patients found a negative correlation between county SVI and influenza vaccine coverage [8]. Notably, the impact of the CDC-SVI theme varied significantly depending on the geographical location. Thus, a theme that is a predictor in one area may not be the same in another. A distinction has been made in several studies examining the uneven distribution of social vulnerability in communities when responding to crises [30–32]. In combination, these studies highlight the variability of influence each theme has in predicting outcomes by location and emphasize the importance of locally tailored interventions.

In our study, minority status was positively associated with incomplete and complete vaccinations in all 17 counties. This suggests that despite the proportion of minorities in a given county, the allocation of resources provided during the pandemic reached a diverse group of Nevadans. Resources included funding, pop-up clinics, mobile units, targeted and tailored messaging, and the use of various methods of communication to support vaccine uptake. On the other hand, housing type and transportation were found to negatively affect vaccination uptake in 15 counties, suggesting that barriers associated with housing and transportation may contribute to place-based disparities. However, statistically insignificant relationships or negative associations are harder to explain with the CDC-SVI and other similar methodologies. Rufat et al. [32] suggest that these relationships can result from the models being a poor indicator of social vulnerability processes, outcome measures that may not adequately reflect the social impacts, or unclearly defined conceptual relationships between social vulnerability and outcomes—further arguing the need for a stronger theoretical basis for selecting validation measures to assess social vulnerability.

Although the CDC-SVI was developed to support planning efforts during disaster relief, it has reliably found its way into broader public health efforts. It has partly done so because of the readily available data and ease of application [33]. However, composite measures have their limitations. For instance, CDC-SVI, and similar indices that rely on census data, are indicator-based and provide a static measure [18,34]. A static measure may not capture the changing dynamics of a community, such as changes to individuals, composition (e.g., demographics), and environment (e.g., development and resources) that occur between data collection periods [18,35]. Considering the complicated and multidimensional nature of social vulnerability, future research should involve developing place-based indicators to account for variable-specific weights [36–38]. Addressing the limitations of existing models may help narrow the gap in our understanding of social vulnerability factors and their potential influence [35].

While our analysis provided an overview of which components of the SVI may be better predictors to gauge vaccination rates, there are a few notable limitations. First, our data were aggregated at the county level and not at a finer area level (e.g., census tract), therefore not allowing us to take a more detailed view of the heterogeneity of the population, which would likely result in more variations [18]. Second, the pandemic resulted in a flood of resources in response to the COVID-19 pandemic, which is not accounted for in the model and may have influenced vaccination rates overall. Lastly, and as noted above, the use of SVI has some limitations. Although it accounts for critical social and demographic factors, it is still a generalized index rather than a customized index specific to COVID-19 to capture all the potential influences on vaccination uptake. Since the SVI methodology is a composite index, it limits the ability to understand which aspects of social vulnerability are more important to vaccination.

## Conclusion

As a result of the unfair and excessive impact on communities that have been made vulnerable, employing vaccine equity efforts is essential to mitigate the impact of long-standing inequities. The CDC-SVI was developed to provide emergency response planners, public health officials, and policymakers with a robust and comprehensive measure of a community’s vulnerability to the impacts of external stressors such as natural disasters and public health emergencies. Although it has its limitations, it certainly is valuable in helping mobilize prevention and mitigation efforts at the community level [34].

Achieving health equity is complex, and it requires adapting intervention models that consider the social, economic, and structural barriers to meet the needs of our communities [38]. Of equal importance is examining or developing alternative or complementary methodologies that help us better capture the influence of social vulnerability factors and understand how specific determinants operate on a local level. As methodology and intervention models are adapted, we will be better equipped to support the development and implementation of public health efforts in communities that need them. Future research should consider the development of social vulnerability models that examine both static and dynamic variables that are weighted to better measure their influence. In addition, more work needs to be done to better understand the mechanism by which social vulnerability measures influence health outcomes.

## References

[1] ATSDR, CDC. At A Glance: CDC/ATSDR Social Vulnerability Index [Internet]. Agency for Toxic Substances and Disease Registry. 2021 [cited 2022 Jul 13]. https://www.atsdr.cdc.gov/placeandhealth/svi/at-a-glance_svi.html.

[2] Pressman AR, Lockhart SH, Shen Z, Azar KMJ. Measuring and Promoting SARS-CoV-2 Vaccine Equity: Development of a COVID-19 Vaccine Equity Index. Health Equity. 2021;5(1):476–83. doi: 10.1089/heq.2021.0047 34316531 PMC8309415

[3] AMA, AAMC. Advancing Health Equity: A Guide to Language, Narrative and Concepts [Internet]. Association of American Medical Colleges Center for Health Justice. 2021 [cited 2023 Aug 28]. https://www.ama-assn.org/system/files/ama-aamc-equity-guide.pdf.

[4] Karaye IM, Horney JA. The Impact of Social Vulnerability on COVID-19 in the U.S.: An Analysis of Spatially Varying Relationships. Am J Prev Med. 2020;59(3):317–25. doi: 10.1016/j.amepre.2020.06.006 32703701 PMC7318979

[5] Nayak A, Islam SJ, Mehta A, Ko YA, Patel SA, Goyal A, et al. Impact of Social Vulnerability on COVID-19 Incidence and Outcomes in the United States. medRxiv. 2020. doi: 10.1101/2020.04.10.20060962 32511437 PMC7217093

[6] Biggs EN, Maloney PM, Rung AL, Peters ES, Robinson WT. The Relationship Between Social Vulnerability and COVID-19 Incidence Among Louisiana Census Tracts. Front Public Health. 2021;8(617976). doi: 10.3389/fpubh.2020.617976 33553098 PMC7856141

[7] CDC. 5 Reasons It Is Important for Adults to Get Vaccinated [Internet]. Centers for Disease Control and Prevention. 2022 [cited 2023 Aug 28]. https://www.cdc.gov/vaccines/adults/reasons-to-vaccinate.html.

[8] Strully KW, Yang TC. County Social Vulnerability and Influenza Vaccine Rates: National and Local Estimates for Medicare Recipients. Am J Prev Med. 2022;62(1):e1–9. doi: 10.1016/j.amepre.2021.06.015 34548222

[9] Abba-Aji M, Stuckler D, Galea S, McKee M. Ethnic/racial minorities’ and migrants’ access to COVID-19 vaccines: A systematic review of barriers and facilitators. J Migr Health. 2022;5:100086. doi: 10.1016/j.jmh.2022.100086 35194589 PMC8855618

[10] Kiefer MK, Mehl R, Costantine MM, Landon MB, Bartholomew A, Mallampati D, et al. Association between social vulnerability and influenza and tetanus-diphtheria-acellular pertussis vaccination in pregnant and postpartum individuals. Am J Obstet Gynecol MFM. 2022;4(3):100603. doi: 10.1016/j.ajogmf.2022.100603 35240346

[11] Bjork A, Morelli V. Immunization Strategies for Healthcare Practices and Providers [Internet]. In: The Pink Book. 14^th^ ed. Washington D.C.: Public Health Foundation; 2021. p. 29–42. https://www.cdc.gov/vaccines/pubs/pinkbook/strat.html.

[12] Jean-Jacques M, Bauchner H. Vaccine Distribution—Equity Left Behind? JAMA. 2021;325(9):829–30. doi: 10.1001/jama.2021.1205 33512381

[13] CDC. COVID-19 Vaccine Equity for Racial and Ethnic Minority Groups [Internet]. Centers for Disease Control and Prevention. 2022 [cited 2023 Aug 28]. https://www.cdc.gov/coronavirus/2019-ncov/community/health-equity/vaccine-equity.html.

[14] Alsan M & Wanamaker M. Tuskegee and the Health of Black Men. Q J Econ. 2018; 133(1):407–455. doi: 10.1093/qje/qjx029 30505005 PMC6258045

[15] Hughes MM, Wang A, Grossman MK, Pun E, Whiteman A, Deng L, et al. County-Level COVID-19 Vaccination Coverage and Social Vulnerability—United States, December 14, 2020–March 1, 2021. MMWR Morb Mortal Wkly Rep. 2021;70(12):431–6. doi: 10.15585/mmwr.mm7012e1 33764963 PMC7993557

[16] Mody A, Bradley C, Redkar S, Fox B, Eshun-Wilson I, Hlatshwayo MG, et al. Quantifying inequities in COVID-19 vaccine distribution over time by social vulnerability, race and ethnicity, and location: A population-level analysis in St. Louis and Kansas City, Missouri. PLoS Med. 2022;19(8):e1004048. doi: 10.1371/journal.pmed.1004048 36026527 PMC9417193

[17] Bruckhaus AA, Abedi A, Salehi S, Pickering TA, Zhang Y, Martinez A, et al. COVID-19 Vaccination Dynamics in the US: Coverage Velocity and Carrying Capacity Based on Socio-demographic Vulnerability Indices in California. J Immigr Minor Health. 2022;24(1):18–30. doi: 10.1007/s10903-021-01308-2 34797451 PMC8603654

[18] Srivastava T, Schmidt H, Sadecki E, Kornides ML. Social vulnerability, disadvantage, and COVID-19 vaccine rationing: A review characterizing the construction of disadvantage indices deployed to promote equitable allocation of resources in the United States. SSRN. 2021. doi: 10.2139/ssrn.3882863

[19] Bauer C, Zhang K, Lee M, Jones M, Rodriguez A, de la Cerda I, et al. Real-time geospatial analysis identifies gaps in COVID-19 vaccination in a minority population. Sci Rep. 2021;11(18117). doi: 10.1038/s41598-021-97416-y 34518570 PMC8437959

[20] Thakore N, Khazanchi R, Orav EJ, Ganguli I. Association of Social Vulnerability, COVID-19 vaccine site density, and vaccination rates in the United States. Healthcare. 2021;9(4):100583. doi: 10.1016/j.hjdsi.2021.100583 34560408 PMC8450273

[21] Lei Y. Hyper Focusing Local Geospatial Data to Improve COVID-19 Vaccine Equity and Distribution Journal of Urban Health. 2021;98(4):453–8. doi: 10.1007/s11524-021-00552-z 34184210 PMC8238383

[22] U.S. Census Bureau. QuickFacts: Nevada [Internet]. U.S. Census Bureau. 2021 [cited 2022 Oct 12]. https://www.census.gov/quickfacts/NV?.

[23] World Population Review. Nevada Population 2022 [Internet]. World Population Review. 2022 [cited 2022 Dec 8]. https://worldpopulationreview.com/states/nevada-population.

[24] U.S. Census Bureau. Race and Ethnicity in the United States: 2010 Census and 2020 Census [Internet]. U.S. Census Bureau. 2021 [cited 2022 Jul 10]. https://www.census.gov/library/visualizations/interactive/race-and-ethnicity-in-the-united-state-2010-and-2020-census.html.

[25] U.S. Census Bureau. DP05 DEMOGRAPHICS AND HOUSING ESTIMATES, 2021 American Community Survey 1-Year Estimates [Internet]. U.S. Census Bureau, American Community Survey Office. 2021 [cited 2024 Jan 1]. https://data.census.gov/table/ACSDP1Y2021.DP05?q=population.

[26] Girnus AC. Census data shows communities of color are the new Nevada [Internet]. Nevada Current. 2021 [cited 2022 Jul 10]. https://www.nevadacurrent.com/2021/08/16/census-data-shows-communities-of-color-are-the-new-nevada/.

[27] ATSDR. CDC SVI Documentation 2020 [Internet]. Agency for Toxic Substances and Disease Registry. 2022 [cited 2023 Aug 28]. https://www.atsdr.cdc.gov/placeandhealth/svi/documentation/SVI_documentation_2020.html.

[28] Fahrmeir L, Kneib T, Lang S, Marx BD. Structured Additive Regression. In: Regression: Models, Methods, and Applications. 2^nd^ ed. Berlin, Heidelberg: Springer; 2021. p. 555–621. doi: 10.1007/978-3-662-63882-8_9

[29] Kindermann R, Snell JL. Markov Random Fields and Their Application. Vol. 1. Providence, RI: Contemporary Mathematics; 1980. https://www.cs.unm.edu/~williams/cs530/conm1-whole.pdf.

[30] Bakkensen LA, Fox-Lent C, Read LK, Linkov I. Validating Resilience and Vulnerability Indices in the Context of Natural Disasters. Risk Analysis. 2017;37(5):982–1004. doi: 10.1111/risa.12677 27577104

[31] Fletcher KM, Espey J, Grossman MK, Sharpe JD, Curriero FC, Wilt GE, et al. Social vulnerability and county stay-at-home behavior during COVID-19 stay-at-home orders, United States, April 7-April 20, 2020. Ann Epidemiol. 2021;64:76–82. doi: 10.1016/j.annepidem.2021.08.020 34500085 PMC8523174

[32] Rufat S, Tate E, Emrich CT, Antolini F. How Valid Are Social Vulnerability Models? Ann Am Assoc Geogr. 2019;109(4):1131–53. doi: 10.1080/24694452.2018.1535887 PMC11611389 39624068

[33] Arling G, Blaser M, Cailas MD, Canar JR, Cooper B, Flax-Hatch J, et al. A Data Driven Approach for Prioritizing COVID-19 Vaccinations in the Midwestern United States. Online J Public Health Inform. 2021;13(1):e5. doi: 10.5210/ojphi.v13i1.11621 33936526 PMC8075414

[34] Wolkin A, Collier S, House JS, Reif D, Motsinger-Reif A, Duca L, et al. Comparison of National Vulnerability Indices Used by the Centers for Disease Control and Prevention for the COVID-19 Response. Public Health Reports. 2022;137(4):803–12. doi: 10.1177/00333549221090262 35514159 PMC9257512

[35] Adepoju OE, Kiaghadi A. Measuring Historic and Longitudinal Social Vulnerability in Disaster-Prone Communities: A Modification to the Centers for Disease Control and Prevention Social Vulnerability Index (CDC-SVI). Disaster Med Public Health Prep. 2023;17:e368. doi: 10.1017/dmp.2023.29 36805737

[36] Spielman SE, Tuccillo J, Folch DC, Schweikert A, Davies R, Wood N, et al. Evaluating social vulnerability indicators: criteria and their application to the Social Vulnerability Index. Natural Hazards. 2020;100(1):417–36. doi: 10.1007/s11069-019-03820-z

[37] Tate E. Social vulnerability indices: A comparative assessment using uncertainty and sensitivity analysis. Natural Hazards. 2012;63:325–47. doi: 10.1007/s11069-012-0152-2

[38] Ogojiaku CN, Allen JC, Anson-Dwamena R, Barnett KS, Adetona O, Im W, et al. The Health Opportunity Index: Understanding the Input to Disparate Health Outcomes in Vulnerable and High-Risk Census Tracts. Int J Environ Res Public Health. 2020;17(16). doi: 10.3390/ijerph17165767 32785046 PMC7459470

